# Bacterial diversity in snow on North Pole ice floes

**DOI:** 10.1007/s00792-014-0660-y

**Published:** 2014-06-21

**Authors:** Aviaja L. Hauptmann, Marek Stibal, Jacob Bælum, Thomas Sicheritz-Pontén, Søren Brunak, Jeff S. Bowman, Lars H. Hansen, Carsten S. Jacobsen, Nikolaj Blom

**Affiliations:** 1 Center for Biosustainability, Technical University of Denmark, Hørsholm, Denmark; 2 Center for Biological Sequence Analysis, Technical University of Denmark, Building 208, 2800 Kongens Lyngby, Denmark; 3 Department of Geochemistry, Geological Survey of Denmark and Greenland (GEUS), Copenhagen, Denmark; 4 Center for Permafrost (CENPERM), University of Copenhagen, Copenhagen, Denmark; 5 Department of Biological Oceanography, University of Washington, Seattle, WA USA; 6 Department of Environmental Science, Aarhus University, Roskilde, Denmark; 7 Department of Plant and Environment, University of Copenhagen, Copenhagen, Denmark

**Keywords:** Polar microbiology, Arctic, Bacterial diversity, Pyrosequencing, Snow

## Abstract

The microbial abundance and diversity in snow on ice floes at three sites near the North Pole was assessed using quantitative PCR and 454 pyrosequencing. Abundance of 16S rRNA genes in the samples ranged between 43 and 248 gene copies per millilitre of melted snow. A total of 291,331 sequences were obtained through 454 pyrosequencing of 16S rRNA genes, resulting in 984 OTUs at 97 % identity. Two sites were dominated by *Cyanobacteria* (72 and 61 %, respectively), including chloroplasts. The third site differed by consisting of 95 % *Proteobacteria*. Principal component analysis showed that the three sites clustered together when compared to the underlying environments of sea ice and ocean water. The Shannon indices ranged from 2.226 to 3.758, and the Chao1 indices showed species richness between 293 and 353 for the three samples. The relatively low abundances and diversity found in the samples indicate a lower rate of microbial input to this snow habitat compared to snow in the proximity of terrestrial and anthropogenic sources of microorganisms. The differences in species composition and diversity between the sites show that apparently similar snow habitats contain a large variation in biodiversity, although the differences were smaller than the differences to the underlying environment. The results support the idea that a globally distributed community exists in snow and that the global snow community can in part be attributed to microbial input from the atmosphere.

**Electronic supplementary material:**

The online version of this article (doi:10.1007/s00792-014-0660-y) contains supplementary material, which is available to authorized users.

## Introduction

The extent of sea ice in the Arctic is diminishing due to climate change (Perovich and Richter-Menge 2009), and it is expected that an ice-free Arctic Ocean will become a reality in coming summers (Wang and Overland 2009). Snow on North Pole ice floes is an example of a virtually unknown microbe-dominated ecosystem undergoing profound change. While the snow microbial community in the high Arctic, Antarctica and Asia has been receiving increasing attention with regard to community structure and biogeographical dispersal of microorganisms (Amato et al. 2007; Liu et al. 2009; Bowers et al. 2009; Larose et al. 2010; Harding et al. 2011; Møller et al. 2013; Hell et al. 2013; Lopatina et al. 2013), there is little knowledge of the diversity of microbes in snow with no direct influence by terrestrial and/or anthropogenic sources. The abundance of bacteria in snow has been found to range from 0.02 × 10^3^ (Harding et al. 2011) to 720 × 10^3^ (Liu et al. 2009) cells per millilitre of melted snow. Recently, it has been shown that microbial abundance in snow may be influenced by anthropogenic sources/activity resulting in higher abundance related to higher input of microorganisms closer to human activity (Lopatina et al. 2013). The North Pole serves as an example of a remote microbial habitat with little impact from human activity.

Whether the bacterial community in snow is globally distributed or if local sources of microorganisms determine the bacterial composition remains unclear (Harding et al. 2011; Larose et al. 2013). Studies from remote environments may help clarify whether snow is merely a reservoir of local sources of organisms or if it hosts a community specific to snow as a habitat, and they may also serve as a substantial contribution to the discussion of the range of dispersal of microorganisms.

Bacteria found in snow may represent recent deposition events, as suggested by studies showing great spatial and temporal variability in the bacterial community composition (Hell et al. 2013; Lopatina et al. 2013) and relation to local environments (Liu et al. 2009; Harding et al. 2011). There are indications, however, that the microbial community in snow is dominated by certain bacteria, including *Betaproteobacteria* that seem to be dominant in most snow habitats followed by other *Proteobacteria, Cyanobacteria, Actinobacteria* and *Bacteroidetes* (Larose et al. 2010; Møller et al. 2013; Hell et al. 2013; Lopatina et al. 2013).

In this paper, we examine the abundance and community composition of microorganisms in snow samples collected in the vicinity of the geographical North Pole during the LOMROG II expedition in 2009, using quantitative PCR and 454 pyrosequencing. We aim to contribute to further insight into the biodiversity of this extreme and remote environment and the possible dispersal mechanisms of microorganisms to snow.

## Materials and methods

### Field sites and sample collection

Samples of snow were collected during the LOMROG II expedition at three different sites in the vicinity of the North Pole (Supplementary Figure S1). Sample A was collected at 85°15′N, 18°18′E on 5 August 2009, sample B was collected on 11 August at 88°55′N, 99°45′E, and sample C on 30 August 2009 at 87°27′N, 16°13′E. All the sites were accessed by helicopter. 120 l of snow was collected from the upper layer of loose snow (max. 20 cm) using a flame-sterilized snow shovel at each site. The snow was placed in 10-l plastic buckets, pre-cleaned with sterile deionized water, and later transferred to sterile 30-l polypropylene bags. The samples were thawed for 36 h in a heated lab on board the ship and, prefiltered through 2.0-µm polypropylene filters and filtered through Steripak GP 0.22-µm polyethersulfone filters (Millipore, Billerica, MA, USA). 30-ml sucrose buffer (0.75 M sucrose) was added to the filters to lyse the DNA. The filters were then capped at both ends with sterile caps, frozen to −20 °C and transported frozen to Copenhagen.

### DNA extraction

Prior to extraction, the Steripak filters were left to thaw in a laminar flow cabinet at room temperature, and the thawed sucrose buffer was refiltered through the filters into sterile 15-ml polypropylene tubes. DNA was then extracted from the filters using the manufacturer’s extraction protocol for the PowerWater Sterivex DNA Isolation Kit (MO BIO Laboratories, Carlsbad, CA, USA), modified for the larger volume of Steripak filters. The volumes of solutions ST1B and ST2 added to the Steripak filters were increased nine-fold to 13.5 ml, the resulting lysate (‘new lysate’) was evacuated from the filters using sterile 50-ml syringes and split evenly into nine glass bead tubes from the PowerWater DNA isolation kit. DNA contained in the original sucrose buffer filtrate was precipitated using the following protocol. The filtrate was mixed with 2-M NaCl and cold ethanol (1:0.1:2 v/v/v filtrate:NaCl:ethanol) in a 50-ml polypropylene tube and incubated at −20 °C for 1 h. The tubes were then centrifuged at 15,000*g* for 15 min, the supernatant was decanted and 1 ml of 70 % ethanol was added. The centrifugation step was then repeated, supernatant decanted, and the pellet was air-dried for 10 min, resuspended in 0.5 ml of DNA-free water, and then added to the glass bead tubes containing the new lysate. In the last elution step of the extraction protocol DNA from 3 tubes was pooled into one, thus resulting in 3 tubes containing 100 µl of DNA extract for each sample. These three subsamples were treated as replicates. This method gave the highest DNA yield compared to a method using the Sterivex protocol only without adding the precipitated DNA from the filtrate and with a method based on proteinase K lysis, as tested on filtered tap water samples (data not shown).

### Quantitative PCR

The abundance of 16S rRNA genes in the extracts was determined using quantitative PCR (qPCR) performed with the PRBA338f and P518r primers (Muyzer et al. 1993) and standards with known quantities of the gene extracted from 1.8 ml of a culture of *E. coli* using an UltraClean microbial DNA isolation kit (MO BIO Laboratories, Carlsbad, CA, USA) following the manufacturer’s protocol. The qPCR was set up under DNA-free conditions in a clean, UV sterilized (3 h on a daily basis) lab with an HEPA-filtered air inlet. The setup was as follows: 20-µl reactions containing 10 µl of SYBR Premix Ex Taq II (TaKaRa, Kyoto, Japan), 0.8 µl of the primers (final concentration 0.4 µM) and 1 µl of template DNA. The reaction was then carried out on a CFX96 Touch qPCR system (Bio-Rad, Hercules, CA, USA). The cycle program was 95 °C for 1 min followed by 50 cycles of 95 °C for 30 s, 30 s at 60 °C and 72 °C for 30 s. The reaction was completed by a final 72 °C elongation step for 6 min. All qPCR reactions were performed in triplicate, and confirmatory melting curve analysis was performed on all qPCR products. Negative controls containing no template were used to assess the contamination potential.

### Pyrosequencing

The diversity of 16S rRNA genes in the snow samples was determined by pyrosequencing. Amplicons of 466-bp flanking the V3 and V4 regions of the 16S rRNA gene were amplified using the primers 341F (5′-CCTAYGGGRBGCASCAG-3′) and 806R (5′-GGACTACNNGGGTATCTAAT-3′) followed by a second PCR where primers with adapters and 10-bp tags were used (Hansen et al. 2012). PCR amplification was performed using 1 × AccuPrime buffer II which contained 0.2 mM dNTP’s, 0.75 U AccuPrime Taq DNA Polymerase High fidelity (Invitrogen, Carlsbad, CA, USA), 0.5 μM of each of the primers, and 1 μl of DNA extract to a total of 25 μl per reaction. PCR was performed with a DNA Engine Dyad Peltier Thermal Cycler (MJ Research, Massachusetts, USA). The cycle conditions were 94 °C for 2 min, 30 cycles of denaturation at 94 °C for 20 s, annealing at 56 °C for 30 s and elongation at 68 °C for 40 s, and a final elongation step at 72 °C for 5 min. The conditions of the second PCR were as the first PCR, except that the number of cycles was reduced to 15 cycles. The PCR products were run on a gel and the appropriate fragments were cut and purified using the Montage DNA Gel Extraction kit (Millipore, Bedford, Massachusetts, USA). The amplified fragments with adapters and tags were quantified using a Qubit fluorometer (Invitrogen, Carlsbad, CA, USA) and mixed in approximately equal concentrations (1 ×  10^7^ copies μl^−1^) to ensure equal representation of each sample. Samples were run on a GS FLX Titanium Pico TiterPlate using a GS FLX Titanium Sequencing Kit XLR70 according to the manufacturer’s instructions (Roche Diagnostics, Indianapolis, IN, USA).

### Bioinformatics

The 454 pyrosequencing data were analyzed using the software package QIIME version 1.7.0 (Caporaso et al. 2010). The data were denoised to remove characteristic sequencing errors for 454 pyrosequencing, specifically faulty additional operational taxonomic units (OTUs) caused by long homopolymers (Reeder and Knight 2010). Chimeras were removed with the USEARCH toolbox version 7.0.1001 UCHIME reference-based chimera detection using the Greengenes database gg_13_5 release downloaded on 13 February 2014 as reference (DeSantis et al. 2006; Edgar et al. 2011). Samples were assigned to multiplex reads with the following default parameters: min. quality score of 25, min. length 200, max. length 1000 and no ambiguous bases and mismatches allowed in primer sequences. De novo OTU picking in QIIME was done by assigning similar sequences to a single OTU at a 97 % similarity threshold. Taxonomy of the OTUs was assigned through QIIME with BLAST (Altschul et al. 1990), the RDP Classifier (Wang et al. 2007) and the QIIME RTAX classifier. Rarefaction curves (Hughes et al. 2001), Shannon indices (Magurran 1988) and richness indices (Chao 1984) were obtained through QIIME. Further analysis of sequences matching cyanobacterial OTUs was done through manually blasting with the NCBI Standard Nucleotide BLAST against the nr/nt database using Megablast (Altschul et al. 1990).

### Statistical analysis

The multivariate data analysis software Canoco 5 (ter Braak and Šmilauer 2012) was used to conduct principal component analysis (PCA) of the relative abundance data to analyze the total variation within the snow dataset, as well as between snow samples and ice and ocean samples. PCA is an unconstrained ordination method that describes the axes of maximum variability in the data and helps discern patterns within datasets. The data were log(*x* + 1) transformed prior to analysis.

## Results and discussion

**Fig. 1:**
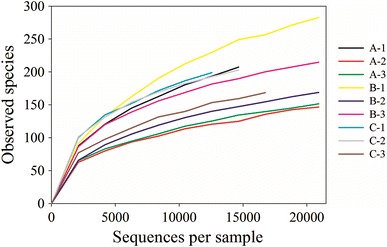
Rarefaction curves for replicates of samples A, B and C showing observed number of species (OTUs at 97 % identity) as a function of sequences per sample

A total of 291,331 16S small subunit ribosomal RNA gene (variable regions 3 and 4) sequences were obtained from the snow samples collected at three sites in the vicinity of the North Pole (Table 1). The average number of sequences per sample was 29,994 and the minimum number was 13,737, which is a hundred- to thousand-fold higher than the numbers obtained from previous snow diversity studies using cloning and Sanger sequencing or bacterial isolation (Carpenter et al. 2000; Amato et al. 2007; Larose et al. 2010). Denoising of the raw sequencing data corrected 182,913 sequences, leaving 269,944 sequences that were subsequently assigned to 984 OTUs. The rarefaction curves (Fig. 1) showing the numbers of observed species as a function of sequences per sample do not reach a plateau, showing that the diversity is still undersampled despite the relatively high number of sequences obtained.

**Table 1:** Overview of the 16S rRNA gene sequence dataset

Total number of sequences	291331
Number of sequences after denoising and chimera removal	269944
Average sequences per sample	29994
Minimum sequences per sample	13737
Maximum sequences per sample	72931
Total number of OTUs	984

Abundances ranged between 43 and 248 16S rRNA genes per ml of melted snow (Table 2), falling in the lower end of what has been shown in other studies with abundances ranging from 20 (Harding et al. 2011) to 720,000 (Liu et al. 2009) cells ml^−1^. A recent study on snow around Russian Antarctic stations showed microbial abundances of 1 × 10^3^ to 46 × 10^3^ cells ml^−1^ and a ten- to hundred-fold increase in DNA content in snow sampled in the proximity of human activity (Lopatina et al. 2013). The low abundances of 16S rRNA genes found in this study indicate that snow from North Pole ice floes is a habitat with low rates of microbial input. Sample A showed the lowest 16S rRNA gene abundance as well as the lowest diversity; however, the highest abundance was determined in sample B and the highest diversity in sample C. This shows that the diversity did not depend on the absolute amount of DNA, or microbial abundance, in the samples alone.

**Table 2:** Diversity indices and 16S rRNA gene abundances in the North Pole snow samples

Site	Shannon index	Chao1 index	Richness	Dominance	16S rRNA gene abundance (gene copies per ml of melted snow)
A	2.226 ± 0.51	314 ± 54	168 ± 33	0.70 ± 0.17	0.043 ± 0.021 × 10^3^
B	3.124 ± 0.22	353 ± 64	222 ± 57	0.31 ± 0.0003	0.248 ± 0.091 × 10^3^
C	3.758 ± 0.35	293 ± 6,7	190 ± 19	0.27 ± 0.01	0.118 ± 0.016 × 10^3^

Mean ± st.dev., *n* = 3

All samples showed a Chao1 index of less than double the number of the observed OTUs (richness) (Table 2), meaning that the observed number of species represents over 50 % of the estimated true number of species. Two other studies have assessed biodiversity in snow by means of 454 pyrosequencing. One study found 333 OTUs per sample in a High Arctic glacier snowpack (Hell et al. 2013) slightly higher than the mean richness of 193 OTUs per sample in our study. Another study showed a mean richness at Station Nord in Northeastern Greenland of 4620 OTUs per sample (Møller et al. 2013). The higher richness detected at Station Nord as well as a Chao1 index of 7841 could be partly attributed to the higher number of sequences obtained in the study. This supports the results of the rarefaction analysis (Fig. 1) showing that, with the number of sequences obtained in present study, the environment of Arctic ice floe snow was still undersampled. The diversity expressed as Shannon indices ranged between 2.23 and 3.76 (Table 2), which is also lower than what was found at Station Nord (5.06–5.60) (Møller et al. 2013). The lower richness and diversity found in snow on North Pole ice floes could be explained by a lower rate of microbial input to this remote site caused by selective elimination of microorganisms from the pool of microbial input from the atmosphere due to the extreme conditions that exist during atmospheric transportation. The higher bacterial diversity found in snow in the proximity of terrestrial and anthropogenic sources is likely to originate from the surrounding habitats, where microbial abundances are higher than compared to environments on the North Pole. This is supported by studies showing similarity of the snow microbial community to the community found in environments adjacent to the sampled snow (Liu et al. 2009; Harding et al. 2011).

**Fig. 2:**
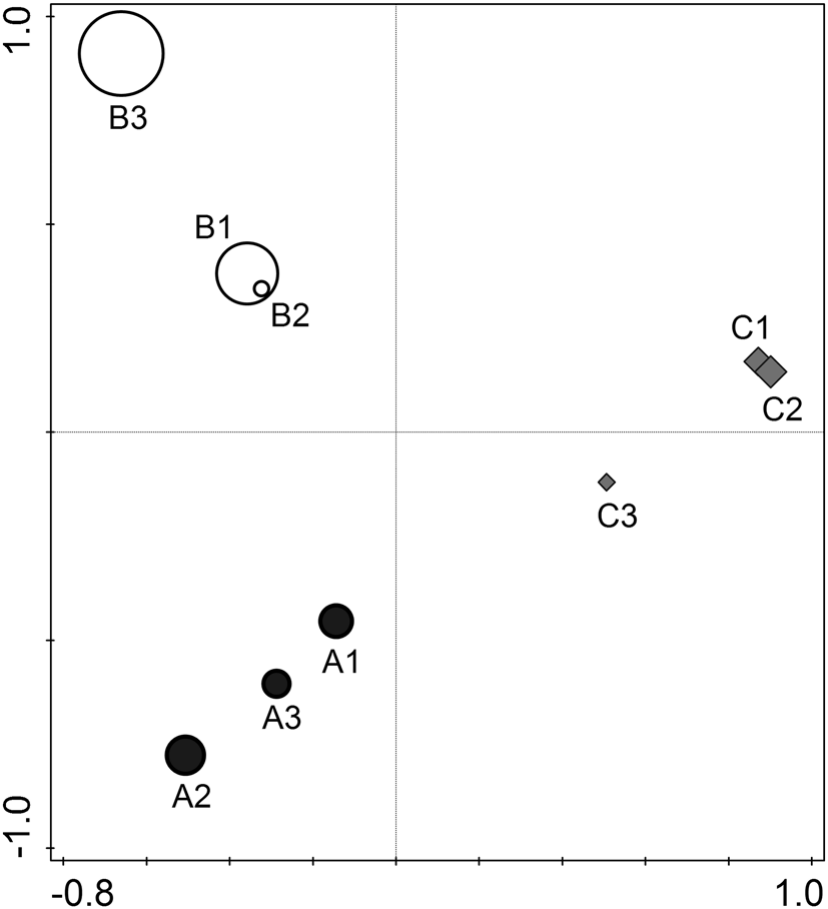
Principal component analysis (PCA) ordination of the microbial diversity data in the snow from the three North Pole sites. The first axis explains 31.8 % of the variation in the data, and the second axis explains 25.9 % of the variation in the data. The size of the *circles* is determined by the richness of the samples

**Fig. 3:**
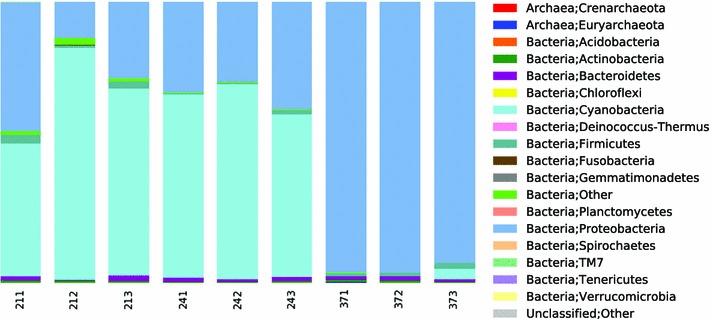
Composition of bacteria in North Pole snow samples A–C at phylum level

The most abundant OTUs in sample A were chloroplasts (69.7 %), *Burkholderiaceae* (7 %) and *Sphingomonas* (4.5 %). Sample B was dominated by diatoms (*Bacillariophyta*, 30.9 %), (26.8 %), chloroplasts and *Shewanella* (11.6 %). The dominant OTUs in sample C were *Pseudoalteromonas* (26.8 %), *Herbaspirillum* (18.4 %) and *Sphingomonas* (7.9 %). The variation in diversity within samples (i.e., between replicates) was smaller than the variation between sites, which is visualised in a principal component analysis plot shown in Fig. 2, where replicates from the same site cluster together. This shows that errors potentially introduced during handling and analyses of the samples are smaller than the real differences between sites. At phylum level, the diversity of samples from site A and B was dominated by *Cyanobacteria*, including chloroplasts, (72 and 61 % respectively), *Proteobacteria* (23 and 35 %), *Firmicutes* (1.9 and 1.2 %) and *Bacteroidetes* (1.1 and 1.5 %) (Fig. 3). Sample C had similar abundances of *Bacteroidetes* (1.3 %) and *Firmicutes* (1.4 %) as in samples A and B, but was otherwise dominated by *Proteobacteria* (95 %) and lacked the high abundance of *Cyanobacteria* (1.4 %). Thus far most studies on snow have found *Proteobacteria* to be dominant, followed by *Bacteroidetes* and *Actinobacteria* (Bowers et al. 2009; Liu et al. 2009; Hell et al. 2013; Lopatina et al. 2013; Møller et al. 2013). *Firmicutes* have also shown to be common in snow (Bowers et al. 2009; Liu et al. 2009; Larose et al. 2010; Hell et al. 2013; Møller et al. 2013), as have *Cyanobacteria* (Bowers et al. 2009; Larose et al. 2010; Harding et al. 2011; Hell et al. 2013; Møller et al. 2013). The results of the present study are in line with previous findings that *Proteobacteria*, *Bacteroidetes, Firmicutes* and *Cyanobacteria* are the most abundant phyla in snow habitats. The similarity found between studies of snow from different locations supports the idea that a core community of bacteria might inhabit the snow habitat globally. Our study shows that the community composition at remote sites with minimum input from terrestrial and anthropogenic sources resembles that of sites in proximity to these sources and that the similarity in communities is, therefore, not a result of input from terrestrial ground, anthropogenic activities or as in our case the underlying environment of ice and ocean.

Samples A and B showed similarity also at the genus level confirming the results at the phylum level (Figure S2). Both samples had a high representation (>1 % of total OTUs) of *Cyanobacteria* and chloroplasts, *Sphingomonas* (*Alphaproteobacteria*), *Burkholderia* (*Betaproteobacteria*), *Pseudomonas* (*Gammaproteobacteria*) and various genera of the *Comamonadaceae* family of *Betaproteobacteria*. Sample C contained a greater variety of sequences related to bacteria known from marine environments than samples A and B, including 4.6 % of *Idiomarina* (Ivanova et al. 2000), 5 % of *Marinobacter* (Gauthier et al. 1992), 3.3 % of *Shewanella* (Hau and Gralnick 2007) and 1.2 % of *Alteromonas* (Baumann et al. 1972). *Shewanella* was the only predominantly marine genus found in samples A and B with over 1 %, representing 11.7 % of sequences in sample B. A higher number of marine-related bacteria suggest that the microbial community at site C had received greater input from the ocean compared to sites A and B at the time of sampling. The marine input may be caused by disruption of the ice and exposure of the snow to aerosols from the ocean, or by warmer or more saline ice, which could result in an easier migration of marine bacteria to the snow cover on the ice surface through brine channels (Ewert and Deming 2013). These factors may also partly account for the higher diversity found in this sample (Table 2). The differences in community composition at the three sites show that the bacterial community in snow is influenced by the surrounding environments, which potentially leads to an increase in biodiversity. It is highly unlikely that any of the three samples could have avoided receiving input from the underlying environment at some time point. The lack of the marine-related bacteria in samples A and B could be explained by these organisms not being able to sustain themselves in the snow, and they may therefore only transiently make up part of the diversity found in sample C.

When pyrosequencing 16S rRNA genes from environmental samples chloroplasts are included due to the evolutionary origin of chloroplasts from ancient *Cyanobacteria* (Raven 1970). Consequently, a number of chloroplast sequences are included in our study. The large amount of sequences from *Cyanobacteria* and chloroplasts found at sites A and B is consistent with previous findings of snow diversity (Bowers et al. 2009; Larose et al. 2010; Liu et al. 2009; Harding et al. 2011; Hell et al. 2013; Møller et al. 2013). While most *Cyanobacteria*/chloroplast OTUs matched uncultured organisms, the ones assigned to known organisms revealed the presence of chloroplasts of marine or halophilic algae, including diatoms (*Synedra*, *Odontella*), cryptophytes (*Rhodomonas*), prasinophytes (*Mamiella, Mantoniella*, *Micromonas*) and xanthophyceans (*Vaucheria*). This further supports that the snow has received input from seawater and/or sea ice.

**Fig. 4:**
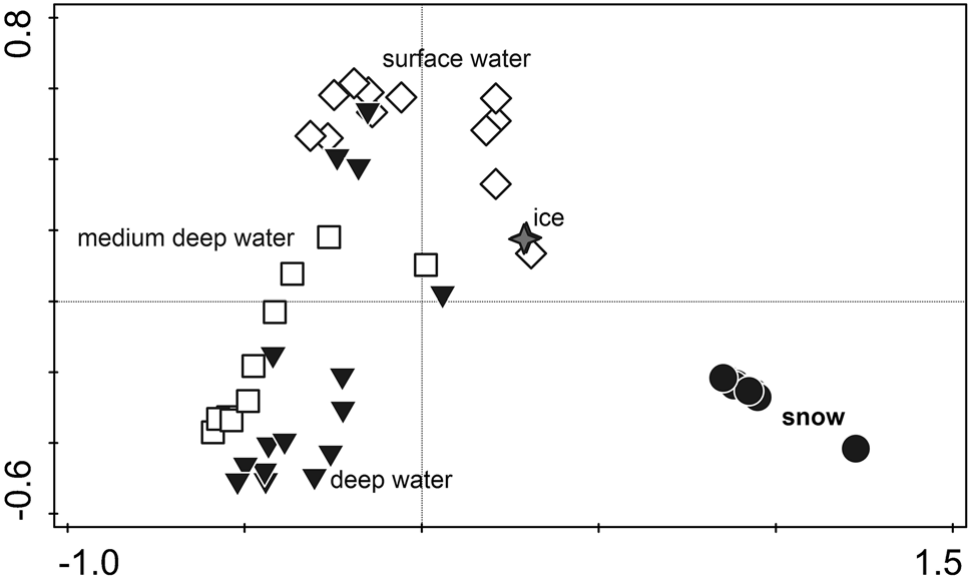
Principal component analysis (PCA) ordination of the microbial diversity data in the snow from the three North Pole sites, ice and ocean water of three depths. The first axis explains 25.9 % of the variation in the data, and the second axis explains 14.8 % of the variation in the data

The community composition in the North Pole snow was compared to the surrounding environment of sea ice and ocean water described in a related study conducted during the LOMROG II expedition (Bowman et al. 2012) using a principal component analysis (Fig. 4). The three snow samples, from sites hundreds of kilometres apart, are more similar to each other than to the underlying sea ice and ocean water. This result again shows that the snow community resembles snow communities at distant sites and, gives further support to the idea of a global microbial community common to snow. These results also suggest that the snow community does not receive a major part of its input of microorganisms from the underlying environment, but more likely from the overlying environment, i.e., the atmosphere. Atmospheric input as a major source of biodiversity is a potential factor behind the global similarities of snow microbial communities.

## Conclusions

This study shows that snow on North Pole ice floes harbors a relatively lower abundance and diversity of microorganisms compared to snow found in the proximity of land and human activity. This indicates that snow at locations remote to terrestrial ground and human activity receives a lower rate of microbial input from the surrounding environment compared to microbial communities in snow at sites closer to terrestrial and anthropogenic sources. Our results also show some importance of local marine sources for the snow biodiversity, although the closer similarities to other snow habitats than to the underlying environment of ice and ocean suggest a global snow community seeded from the atmosphere.

## Electronic supplementary material

Below is the link to the electronic supplementary material.

Supplementary material 1 (JPEG 46 kb)

Supplementary material 2 (PNG 7 kb)

Supplementary material 3 (PDF 5635 kb)
