# Supplementary figures and images for: Bacterial diversity in snow on North Pole ice floes
Source: Extremophiles. 2014 Jun 21;18(6):945–51. doi: 10.1007/s00792-014-0660-y (PMC4196135; doi:10.1007/s00792-014-0660-y)

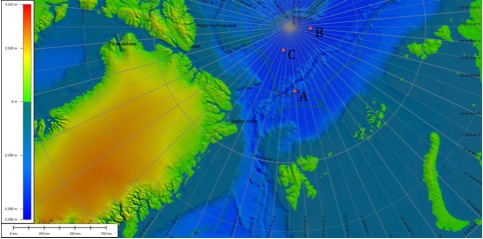

Supplement: Supplementary file 1 — Supplementary material 1 (JPEG 46 kb) [file 792_2014_660_MOESM1_ESM.jpg]

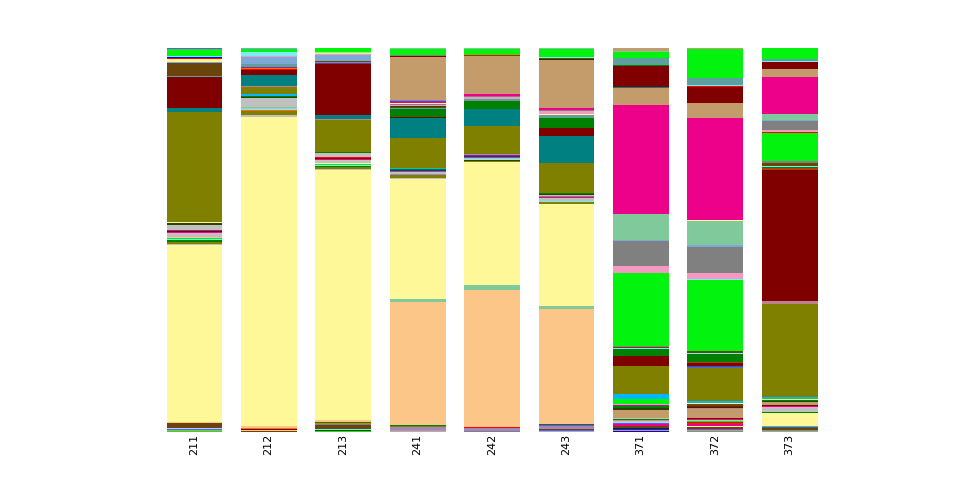

Supplement: Supplementary file 2 — Supplementary material 2 (PNG 7 kb) [file 792_2014_660_MOESM2_ESM.png]
